# Impact of Preoperative Total Knee Arthroplasty on Radiological and Clinical Outcomes of Spinal Fusion for Concurrent Knee Osteoarthritis and Degenerative Lumbar Spinal Diseases

**DOI:** 10.3390/jcm10194475

**Published:** 2021-09-28

**Authors:** Hong Jin Kim, Jae Hyuk Yang, Dong-Gune Chang, Seung Woo Suh, Hoon Jo, Sang-Il Kim, Kwang-Sup Song, Woojin Cho

**Affiliations:** 1 Department of Orthopedic Surgery, Inje University Sanggye Paik Hospital, College of Medicine, Inje University, Seoul 01757, Korea; hongjin0925@naver.com (H.J.K.); hunis121@gmail.com (H.J.); 2 Department of Orthopedic Surgery, Korea University Guro Hospital, College of Medicine, Korea University, Seoul 08308, Korea; kuspine@naver.com (J.H.Y.); spine@korea.ac.kr (S.W.S.); 3 Department of Orthopedic Surgery, College of Medicine, The Catholic University of Korea, Seoul 06591, Korea; sang1kim81@gmail.com; 4 Department of Orthopedic Surgery, Chung-Ang University Hospital, College of Medicine, Chung-Ang University, Seoul 06973, Korea; ksong70@cau.ac.kr; 5 Department of Orthopedic Surgery, Albert Einstein College of Medicine, Montefiore Medical Center, Bronx, NY 10467, USA; woojinchomd@aol.com

**Keywords:** spinal fusion, total knee arthroplasty, lumbar lordosis, sagittal spinopelvic parameters, clinical outcome

## Abstract

Concurrent knee osteoarthritis (KOA) and degenerative lumbar spinal disease (LSD) has increased, but the total knee arthroplasty (TKA) effect on degenerative LSD remains unclear. The aim of this study was to retrospectively analyze to compare radiological and clinical outcomes between spinal fusion only and preoperative TKA with spinal fusion for the patients with concurrent KOA and degenerative LSD. A total of 72 patients with concurrent KOA and degenerative LSDs who underwent spinal fusion at less than three levels were divided in two groups: non-TKA group (*n* = 50) and preoperative TKA group (*n* = 22). Preoperative lumbar lordosis (LL) was significantly lower in the preoperative TKA group than the non-TKA group (*p* < 0.05). Significantly higher preoperative pelvic incidence (PI), PI/LL mismatch, and pelvic tilt (PT) occurred in preoperative TKA group than non-TKA group (all *p* < 0.05). There was significant improvement of postoperative Oswestry Disability Index and leg Visual Analog Scale in the preoperative TKA group (all *p* < 0.01). Preoperative TKA could be a benefit for in proper correction of sagittal spinopelvic alignment by spinal fusion. Therefore, preoperative TKA could be considered a preceding surgical option for patients with severe sagittal spinopelvic parameters in concurrent KOA and degenerative LSD.

## 1. Introduction

With aging populations, the prevalence of concurrent degenerative musculoskeletal condition has increased, which has impacted global disease burden [1]. Degenerative lumbar spinal diseases (LSDs) are one of the most common musculoskeletal conditions caused by degenerative change in spinal joints, intervertebral disks, and ligament flavum, which can lead to load-bearing abnormalities including spinal stenosis, spondylolisthesis, herniated intervertebral disk, and degenerative lumbar scoliosis that are associated with adult spinal deformity [1,2,3]. Knee osteoarthritis (KOA) shares similar clinical presentations with degenerative LSD and is treated by total knee arthroplasty (TKA) in severe cases [4]. Patients frequently have concurrent KOA and degenerative LSD, and it is not uncommon that both disorders are severe enough to require surgical treatment [1,4].

Both degenerative diseases located in spine and knee have an effect on spinal alignment, which necessary for harmonious balances from upright posture to ambulation [3]. In particular, sagittal spinopelvic imbalances occurred in degenerative diseases in spine, as a result of the compensatory mechanism from loss of lordosis, pelvic retroversion, and knee flexion [2,3,4,5]. Furthermore, the stiffness of degenerative knee was reported to affect spinal malalignment because postural equilibrium was harmonized with coordinated movement of spine, hip, and knee [6]. Although knee stiffness significantly impacts on the biomechanical effect of spinal balances, few studies reported on the relationship between TKA and such malalignments to date [6,7,8]. In addition, there is lack of information on how resolution of knee stiffness by TKA affects spinal alignment. Furthermore, there are few studies on the effect of spinal balances between spine fusion and resolution of knee stiffness.

TKA is a well-established surgical treatment, as well as an efficacious way to decrease pain and improve functions for patients with KOA [9]. Surgical treatment of degenerative LSDs and KOA demonstrate uniformly favorable clinical outcomes, according to mid-term to long-term follow-up studies [10,11,12]. However, the effect of certain comorbidities on degenerative LSDs remains unclear. To date, decision-making for fusion surgery or TKA combines the patient’s preferences and surgeons’ assessment of the severity of both diseases [4]. When concurrent KOA and degenerative LSDs are of equally severe grade, there is insufficient evidence for the optimal order of surgical treatment [4]. To the best of our knowledge, there have been very few reports that performed a comparative analysis of spinal fusion in patients with and without TKA. Therefore, this study aimed to analyze the impact of TKA by comparing the clinical and radiological outcomes of spinal fusion for patients with concurrent severe KOA and degenerative LSDs.

## 2. Materials and Methods

This study was performed through retrospective comparative analysis at a single institute where spinal fusion and TKA were routinely performed. The concept and procedures of the study were approved by our institutional review board. All spinal fusion surgery procedures (posterior decompression with posterior lumbar interbody fusion and/or posterior lateral fusion with resected local bone graft and cages) and TKA were performed by senior surgeons (a spine surgeon and a knee surgeon) with vast experience in performing standard surgeries. The patients with hip and/or ankle osteoarthritis above moderate grade or patients who underwent hip arthroplasty, ankle fusion, ankle arthroplasty, and revision TKA were excluded from this study. The medical records data of 122 patients who underwent TKA before spinal fusion or underwent spinal fusion at less than three levels due to degenerative LSDs concurrent with KOA (more than Kellgren-Lawrence grade III) were collected from 2013 to 2018. A total of 72 patients were included, excluding loss to follow-up (*n* = 17) and those who underwent TKA during the postoperative follow-up period of spinal fusion (*n* = 21). The minimum interval between TKA and spinal fusion was set to one-year in consideration of TKA-related pain for at least 6 months. The patients were divided into two groups as follows: the non-TKA group (*n* = 50, patients who underwent spinal fusion only) and the preoperative TKA group (*n* = 22, patients who underwent spinal fusion after TKA)

All patient data were collected from the hospital database and retrospectively analyzed in 2021. Demographic and operative variables included age, height, weight, body mass index (BMI), bone mineral density (BMD), symptom duration, main diagnosis of LSD, spinal stenosis grade on magnetic resonance imaging (MRI), fusion levels, and Kellgren-Lawrence grade. Spinal stenosis grade on MRI was measured by qualitative grading system according to axial MRI on T2-weighted images [13]. Kellgren-Lawrence grade on plain radiograph of knee was evaluated as follows: grade I (doubtful joint space narrowing and possible osteolytic lipping), grade II (definite osteophytes and possible joint space narrowing), grade III (multiple osteophytes, definite joint space narrowing, sclerosis, possible bony deformity), and grade IV (large osteophytes, marked narrowing of joint space, severe sclerosis, and definite deformity of bone contour) [9].

Radiological variables included regional, global, coronal, and sagittal spinopelvic parameters preoperatively, immediate postoperatively (within 2 weeks), and at postoperative 2-year follow-up after spinal fusion. Lumbar lordosis (LL), thoracic kyphosis (TK), and cervical lordosis (CL) were collected as regional parameters. Sagittal vertical axis (SVA) and T1 pelvic angle (TPA) were collected as global parameters. Coronal parameters were measured by Cobb’s angle reflecting local alignment and coronal balance reflecting global alignment. Sagittal spinopelvic parameters included pelvic incidence (PI), PI/LL mismatch, pelvic tilt (PT), and sacral slope. Regarding clinical outcomes, Oswestry Disability Index (ODI) and Visual Analog Scale (VAS) of the leg and back were used for clinical evaluation preoperatively, immediate postoperatively (discharge from hospital) and at postoperative 6-month follow-up after spinal fusion.

Statistical analysis was performed using SPSS Statistics for Windows, version 21.0 (IBM Corp., Armonk, NY, USA). A normal distribution was confirmed by Kolmogorov–Smirnov test. Regarding continuous variables, student-t-test and Mann–Whitney test were used for parametric data and non-parametric data, as appropriate. Regarding categorical variables, chi-square test and Fisher-exact test were used for parametric and non-parametric data, as appropriate. In the case of variables with negative or positive values based on the measured reference point, such as coronal balance and SVA, statistical comparisons of groups required converting negative numbers to positive numbers because it was necessary to statistically analyze differences from a reference point. Statistical significance was set at *p* < 0.05.

## 3. Results

### 3.1. Demographic Data

All demographic, clinical, and operative data, including sex, age, body mass index (BMI), bone mineral density (BMD), symptom duration, main diagnosis of LSDs, spinal stenosis grade on MRI, fusion levels, and Kellgren-Lawrence grade were summarized in Table 1. In preoperative TKA group, mean interval between TKA and spinal fusion was 1.2 years. The mean age in the non-TKA and preoperative TKA groups was 68.4 years and 72.1 years, respectively (*p* = 0.110). Mean BMI in the non-TKA and preoperative TKA groups was 26 and 25.5, respectively (*p* = 0.602). Mean BMD in non-TKA and preoperative TKA groups was −0.7 and −1.1 at the spine as well as −1.1 and −1.4 at the femur. There were no significant differences in BMD of the spine and femur between the two groups (*p* = 0.696, *p* = 0.284). In total, 58% and 59.2 of patients had a symptom duration of more than 5 years in the non-TKA and preoperative TKA groups, respectively. A severe grade of spinal stenosis was presented in 52% and 54.5% of the non-TKA and preoperative TKA groups, respectively. The fusion levels in non-TKA and preoperative TKA group were not significant different (*p* = 0.409). Spondylolisthesis was presented in 26% of the non-TKA group and 45% of the preoperative TKA group for the main diagnosis of LSDs. All KOA were bilateral, which showed more than Kellgren-Lawrence grade III. There were no significant differences in demographic and operative data between the two groups (Table 1).

### 3.2. Radiological Outcomes

Regarding the regional and global parameters of radiological outcomes, preoperative LL was significantly lower in the preoperative TKA group (32°) than the non-TKA group (23°) (*p* = 0.045). The 2-year follow-up LL was lower in the non-TKA group (35.3°) than the preoperative TKA group (27.1°) with statistical significance (*p* = 0.041). Preoperative SVA was 51.6 mm in the non-TKA group and 72.5 mm in the preoperative TKA group, with no significance (*p* = 0.066). Immediate postoperative (40 mm, 47.2 mm) and 2-year follow-up (41.2 mm, 47 mm) SVA in non-TKA and preoperative TKA groups was distributed within an age-adjusted target (about 54.5 mm from 65 to 74 years) with no significance (*p* = 0.455, 0.561) [3]. All TPAs were greater than 20° and those in the preoperative TKA group were higher than non-TKA group, but statistical difference was not significant. Regional and global parameters demonstrated worse outcomes in the preoperative TKA group than the non-TKA group. Only the preoperative and 2-year follow-up LL showed statistically significant differences (Table 2).

Regarding the coronal parameters, Cobb’s angle preoperatively, immediate postoperative, and at 2-year follow-up was within 10° in both groups (all *p* > 0.05). All coronal balance values preoperatively, immediate postoperatively and at 2-year follow-up evaluations were within 20 mm and showed statistical insignificance between the two groups (all *p* > 0.05). For sagittal spinopelvic parameters, preoperative PI was significantly higher in the preoperative TKA group (62.8°) than the non-TKA group (53.5°) (*p* = 0.041). However, after spinal fusion, there were no significance differences between immediate postoperative (*p* = 0.398) and 2-year follow-up (*p* = 0.729) PI. All values of PI/LL mismatch were more than 11°. Preoperative PI/LL mismatch was significantly higher in the preoperative TKA group (39.8°) than the non-TKA group (21.5°) with statistical significance (*p* = 0.013). However, there were no significant difference observed in immediate postoperative (*p* = 0.286) and 2-year follow-up (*p* = 0.265) PI/LL mismatch. PT was greater at more than 22° and was higher in the preoperative TKA group (30.7°) than the non-TKA group (24°). Only preoperative PT showed a statistically difference (*p* = 0.011). All sacral slopes were greater in the preoperative TKA group than in the non-TKA group but without statistical significance (all *p* > 0.05) (Table 3).

### 3.3. Clinical Outcomes

ODI and VAS were used for assessing clinical outcomes preoperatively, immediate postoperatively, and at 6-month follow-up. The mean preoperative ODI was significantly worse in the preoperative TKA group (62.4) than the non-TKA group (50.4) (*p* = 0.001). However, after spinal fusion, the mean immediate postoperative ODI was 45.4 in the non-TKA group and 37.6 in the preoperative TKA group (*p* = 0.008). Mean 6-month follow-up ODI was 45.8 in the non-TKA group and 34. 1 in the preoperative TKA group (*p* < 0.001). Mean preoperative VAS of the back was 7.57 in the non-TKA group and 8.44 in the preoperative TKA group. Mean immediate postoperative VAS of the back was 4.00 in the non-TKA group and 4.44 in the preoperative TKA group. Mean 6-month follow-up VAS of the back was 3.19 in the non-TKA group and 3.33 in the preoperative TKA group. None of these back VAS values were significantly different between groups (all *p* > 0.05). Preoperative VAS of the leg was close to 7.2 in the non-TKA group and 7.3 in the preoperative TKA (*p* = 0.965). Mean immediate postoperative VAS of the leg was 6.1 in the non-TKA group and 3 in the preoperative TKA group (*p* < 0.001). Six-month follow-up VAS of the leg was 6 in the non-TKA group and 2.7 in the preoperative TKA group, a significant difference (*p* < 0.001) (Table 4).

The ODI differences between preoperative and immediate postoperative was 5.0 ± 4.7 in non-TKA and 24.9 ± 6.2 in preoperative TKA with statistical significance (*p* < 0.001). VAS leg differences between preoperative and immediate postoperative was 1.0 ± 0.9 in non-TKA and 4.3 ± 1.9 in preoperative TKA with statistical significance (*p* < 0.001). However, ODI differences and VAS leg differences between immediate postoperative and 6-month follow-up showed not statistical insignificance (*p* = 0.780).

## 4. Discussion

Degenerative diseases including osteoarthritis and spinal stenosis are serious public health concerns globally because of the severe pain and disability they cause [14]. Specifically, lower back pain and osteoarthritis were the first ranked and 12th ranked, respectively, global burden of diseases that cause disability from a systemic analysis in 2016 [15]. Moreover, these chronic conditions lead to multi-morbidity, which limit function and cause pain and disability [14,15]. However, the impact of multi-morbid conditions has not been extensively studied yet [14]. In an arthroplasty study, the impact of total hip arthroplasty in spinal fusion was reported in hip-spine syndrome, but there is a relative lack of evidence for that of TKA [4]. Therefore, this study aimed to identify the impact of preoperative TKA in spinal fusion for patients with concurrent severe KOA and degenerative LSD.

Regarding preoperative radiological parameters, our results showed that LL and sagittal spinopelvic parameters were worse in the TKA group. There were attempts to elucidate the association between radiological factors of the spine and flexibility of the knee [6,16,17]. Flexion contracture of the knee was associated with not only loss of LL, but also poor sagittal spinopelvic parameters [16,17]. Kim et al. suggested that lumbar flexibility is important for spinal and lower limb alignment following TKA [7]. However, the studies reported that removal of flexion contracture by TKA could not compensate for sagittal global imbalances [5,6]. The results have similar preoperative aspects of worse LL and sagittal spinopelvic parameters, which support the finding that TKA does not compensate for these parameters. Our results suggest the patients that require both TKA and spinal fusion have relatively worse preoperative radiological outcomes in LL and sagittal spinopelvic parameters. Therefore, sagittal spinopelvic parameters could consider one of the factors for surgical decision-making in the patients with severe KOA and degenerative LSDs.

The pelvic morphology, which is influenced by sagittal malalignment, was significantly different in elderly patients with concurrent KOA and degenerative LSDs compared to patients with LSD only [18]. Increased sagittal malalignment with a lack of LL was caused by double-level listhesis (i.e., spondylolisthesis and/or retrolisthesis) and greater knee flexion [19]. Although decompression with short-segment fusion at less than three levels can yield improvement of clinical outcomes, corrective lumbar surgery alone may be insufficient for radiological outcomes because of greater pelvic retroversion (high PT) and, worse sagittal spinopelvic alignment [20,21]. Kohno et al. reported that surgical strategies in concurrent degenerative knee and LSDs may be necessary to restore sagittal spinopelvic alignment, followed by decreased pelvic retroversion [18]. In our study, patients with preoperative TKA exhibited greater pelvic retroversion than patients with KOA, and more often required fusion surgery for correction of sagittal spinopelvic alignment. The optimal values of sagittal spinopelvic parameters that need to be corrected was under-estimated by compensatory mechanism of spine from knee stiffness in non-TKA group. Therefore, preoperative TKA could be a benefit for in proper correction of sagittal spinopelvic alignment by spinal fusion.

Schwab et al. showed a PI/LL mismatch that reflected the disharmony between spine and pelvis correlate with increase in ODI [22]. From our result, the preoperative TKA group (i.e., the patients who needs to both spinal fusion and TKA) showed worse ODI values. Because TKA with worse sagittal spinopelvic parameters is associated with poor range of motion, it led to dissatisfaction and did not improve disability [6]. For significant improvement of ODI in the TKA group, preoperative TKA may have contributed to more vigorous activity by resolution of neurogenic claudication. The most important thing in our study was that complementing compensatory mechanisms by preoperative TKA gave a chance for better correction of sagittal spinopelvic parameters, which has a significant impact on improving disability. The value of ODI reflects pain as well as activities of daily living affected by knee discomfort [4]. Lee et al. reported that the presence of preoperative KOA and multi-level fusion were poor prognostic factors in lumbar spinal surgery, and Lee et al. also showed worse ODI scores in the patients who underwent TKA before spinal fusion on retrospective case analysis [23]. However, considering that our study included patients with spinal fusion at less than three levels, preoperatively worse spinopelvic sagittal parameters as well as lower lumbar lordosis contributed to a higher ODI level in the preoperative TKA group compared to the non-TKA group [24]. If the case of long-level spinal fusion and instrumentation, this can clearly affect balancing and lumbar spine alignment by nonunion and/or instrumentation failure. Therefore, in order to minimize this effect and evaluate the impact of preoperative TKA, we assessed only the patents who underwent spinal fusion at less than three-level (i.e., short-level fusion). Preoperative TKA in spinal fusion at less than three levels could be helpful for predicting disability and pain in the case of worse sagittal spinopelvic parameters.

Lower back pain is affected by various factors, and has a broad spectrum of symptoms that requires differential diagnosis based on degenerative, congenital, and traumatic causes [25]. Escobar et al. reported the preoperative absence of lower back pain in TKA as a predictor of a good quality of life in a multi-center prospective study conducted in 2007 [26]. Pivec et al. also suggested that the presence of spinal stenosis was associated with worse clinical outcomes following TKA [27]. However, little is known about the clinical relevance between back pain and preoperative TKA for fusion surgery in patients with KOA. In our study, back VAS was not significantly different between the two groups, which indicates that preoperative TKA in spinal fusion does not seem to have much impact on lower back pain. Preoperative TKA in spinal fusion showed better clinical outcomes in terms of leg VAS, which means significantly improved pain. Lumbar radiculopathy by nerve root compression from L3 to L5 is a typical clinical presentation of spinal stenosis, which share the same portion in anterior knee pain by joint degeneration [28]. Furthermore, the origin of pain from knee and/or spine could be impact on determining clinical outcomes [29]. Therefore, preoperative TKA in the case of short-level spinal fusion significantly impacts improvement by eradicating the pain source.

There were several limitations to our study. First, the number of patients was relatively small and we used a retrospective design. Future trials would be needed by large sample in multicenter study and/or meta-analysis. Secondly, this study did not reflect the morphology and clinical scales of the knee. It also included the limitation of being a retrospective study, which suggests the need to evaluate radiological factors and clinical function of the knee in future trials. However, our study focused on comparing radiological factors, function, and pain measures limited to the spine. Large multi-center prospective studies should be needed to perform to confirm our results. Nonetheless, our study suggested that preoperative TKA in spinal fusion (less than three levels) have significantly impact on lumbar radiculopathy and disability.

## 5. Conclusions

Preoperative TKA could be a benefit for in proper correction of sagittal spinopelvic alignment by spinal fusion. Therefore, preoperative TKA could be considered a preceding surgical option for patients with severe sagittal spinopelvic parameters in concurrent KOA and degenerative LSD.

## Figures and Tables

**Table 1:** Demographic and operative data for spinal fusion only and preoperative TKA with spinal fusion groups.

Variables	Non-TKA (*n* = 50)	Preoperative TKA (*n* = 22)	*p*-Value
Sex (M:F)	9:41	3:18	0.268 ^†^
Age (years)	68.4 ± 7.9 *	72.1 ± 8.1 *	0.110
Height (cm)	155.4 ± 6.3 *	155.9 ± 5.1 *	0.787
Weight (kg)	62.7 ± 8.9 *	62.0 ± 10.1 *	0.786
BMI (kg/m^2^)	26.0 ± 3.5 *	25.5 ± 3.6 *	0.602
BMD (T-score)			
Spine	−0.7 ± 1.0 *	−0.8 ± 1.2 *	0.695
Femur	−1.1 ± 1.0 *	−1.4 ± 0.9 *	0.284
Symptom duration (*n*)			0.303 ^†^
6 months–1 year	10	2	
1–5 years	11	7	
>5 years	29	13	
Main diagnosis of LSD (*n*)			0.205 ^†^
Spinal stenosis	37	12	
Spondylolisthesis with spinal stenosis	13	10	
Spinal stenosis grade on MRI (*n*)			0.806 ^†^
Moderate	14	6	
Moderate to severe	10	4	
Severe	26	12	
Fusion levels (*n*)			0.409 ^†^
1 level	22	12	
2 levels	28	10	
Kellgren-Lawrence grade (*n*, Right:Left)			
Grade III	28:30	-	
Grade IV	22:20	-	

*p* < 0.05 is significant. * All values are expressed as mean ± standard deviation. *p* values were calculated by independent t-test for parametric data and Mann Whitney U test for non-parametric data. ^†^ *p*-values were calculated by chi-square test for parametric data and Fisher’s exact test for non-parametric data. *n* = number; TKA = Total knee arthroplasty; M = Male; F = Female; BMI = Body mass index; BMD = Bone mineral density; LSDs = Lumbar spinal diseases; MRI = Magnetic resonance imaging.

**Table 2:** Comparison of regional and global parameters between spinal fusion only and preoperative TKA with spinal fusion groups.

Variables	Non-TKA (*n* = 50)	Preoperative TKA (*n* = 22)	*p*-Value
Regional parameters			
Lumbar lordosis (°)			
Preoperative	32.0 ± 16.0	23.0 ± 13.5	0.045
Immediate postoperative	34.1 ± 13.5	29.9 ± 12.6	0.274
2-year follow-up	35.3 ± 13.7	27.1 ± 13.6	0.041
Thoracic kyphosis (°)			
Preoperative	28.9 ± 12.6	24.4 ± 11.7	0.213
Immediate postoperative	28.9 ± 11.0	27.4 ± 9.5	0.643
2-year follow-up	28.9 ± 10.9	26.7 ± 11.4	0.5
Cervical lordosis (°)			
Preoperative	20.9 ± 10.2	18.1 ± 7.5	0.326
Immediate postoperative	21.0 ± 10.2	19.3 ± 8.6	0.572
2-year follow-up	21.6 ± 10.1	18.6 ± 7.5	0.28
Global parameters			
Sagittal Vertical Axis (mm)			
Preoperative	51.6 ± 30.8	72.5 ± 56.4	0.066
Immediate postoperative	40.0 ± 32.5	47.2 ± 30.9	0.455
2-year follow-up	41.2 ± 34.0	47.0 ± 30.9	0.561
T1 pelvic angle (°)			
Preoperative	26.3 ± 7.6	28.9 ± 7.3	0.247
Immediate postoperative	24.0 ± 7.0	22.1 ± 6.0	0.343
2-year follow-up	24.6 ± 7.3	22.6 ± 2.4	0.425

Data represent mean ± standard deviation values for each group. In the case of the sagittal vertical axis, the statistical analysis between groups was performed by converting negative numbers to positive numbers to analyze how the difference from the reference point. *p*-values were calculated by independent t-test for parametric data and Mann Whitney U test for non-parametric data. Significant differences were accepted for *p* < 0.05. *n* = number; TKA = Total knee arthroplasty.

**Table 3:** Comparison of coronal and sagittal spinopelvic parameters between spinal fusion only and preoperative TKA with spinal fusion groups.

Variables	Non-TKA (*n* = 50)	Preoperative TKA (*n* = 22)	*p*-Value
Coronal parameters			
Cobb’s angle (°)			
Preoperative	7.4 ± 5.4	8.5 ± 9.6	0.551
Immediate postoperative	6.3 ± 5.4	6.1 ± 5.0	0.887
2-year follow-up	6.6 ± 5.9	5.9 ± 5.3	0.67
Coronal balance (mm)			
Preoperative	9.1 ± 8.2	9.8 ± 8.8	0.783
Immediate postoperative	6.2 ± 4.9	9.7 ± 10.3	0.07
2-year follow-up	5.4 ± 4.4	12.9 ± 28.9	0.093
Sagittal spinopelvic parameters			
Pelvic incidence (°)			
Preoperative	53.5 ± 16.2	62.8 ± 13.1	0.041
Immediate postoperative	56.9 ± 16.9	61.3 ± 21.0	0.398
2-year follow-up	61.0 ± 16.2	59.5 ± 15.0	0.729
PI/LL mismatch			
Preoperative	21.5 ± 25.8	39.8 ± 21.7	0.013
Immediate postoperative	23.7 ± 10.3	31.1 ± 15.6	0.286
2-year follow-up	25.7 ± 20.1	32.3 ± 22.2	0.265
Pelvic tilt (°)			
Preoperative	24.0 ± 8.4	30.7 ± 10.2	0.011
Immediate postoperative	26.4 ± 9.9	27.8 ± 9.2	0.609
2-year follow-up	29.3 ± 11.7	29.6 ± 11.5	0.935
Sacral slope (°)			
Preoperative	29.5 ± 8.0	32.1 ± 9.6	0.286
Immediate postoperative	30.5 ± 7.5	33.4 ± 17.7	0.349
2-year follow-up	31.7 ± 6.8	29.8 ± 7.5	0.37

Data represent mean ± standard deviation values for each group. In the case of coronal balance, the statistical analysis between groups was performed by converting negative numbers to positive numbers to analyze how the difference from the reference point. *p* values were calculated by independent t-test for parametric data and Mann Whitney U test for non-parametric data. Significant differences were accepted for *p* < 0.05. *n* = number; TKA = Total Knee Arthroplasty; PI/LL mismatch = Pelvic incidence minus lumbar lordosis.

**Table 4:** Comparison for clinical outcomes between spinal fusion only and preoperative TKA with spinal fusion.

Clinical Outcomes	Non-TKA (*n* = 50)	Preoperative TKA (*n* = 22)	*p*-Value
ODI			
Preoperative	50.4 ± 9.0	62.4 ± 5.5	0.001
Immediate postoperative	45.4 ± 10.7	37.6 ± 5.3	0.008
6-month follow-up	45.8 ± 8.8	34.1 ± 4.7	<0.001
VAS Back			
Preoperative	7.6 ± 1.6	8.4 ± 1.2	0.193
Immediate postoperative	4.0 ± 0.8	4.4 ± 1.1	0.642
6-month follow-up	3.2 ± 0.8	3.3 ± 1.0	0.79
VAS Leg			
Preoperative	7.2 ± 1.7	7.3 ± 2. 0	0.965
Immediate postoperative	6.1 ± 1.5	3.0 ± 0.7	<0.001
6-month follow-up	6.0 ± 1.1	2.7 ± 0.7	<0.001

Data represent mean ± standard deviation values for each group. *p*-values were calculated by independent t-test for parametric data and Mann–Whitney U test for non-parametric data. Significant differences were accepted for *p* < 0.05. *n* = number; TKA = Total knee arthroplasty; ODI = Oswestry Disability Index; VAS = Visual Analog Scale.

## Data Availability

Data collected for this study, including individual patient data, will not be made available.
